# ER‐Localized Deadenylase *PNLDC1* Suppresses Colorectal Cancer Progression by Targeting the mRNA Decay of *TUBB4B*

**DOI:** 10.1002/exp2.70222

**Published:** 2026-09-21

**Authors:** Lexin Liu, Yuelin Guan, Yuzhou Qin, Guiping Chen, Guozhen Wang, Xiang Yan, Xue Li, Lei Sun, Wei Yu, Shi Ouyang, Zhichao Ma, Zihao Xu, Zhongkai Cao, Fan Yu, Tong Zhou, Ziyou Luo, Xiangjun Chen, Yongbin Yan, Lidan Hu

**Affiliations:** ^1^ Department of Urology, Children's Hospital, Zhejiang University School of Medicine National Clinical Research Center for Children and Adolescents’ Health and Diseases Hangzhou China; ^2^ School of Basic Medical Sciences and Forensic Medicine Hangzhou Medical College Hangzhou Zhejiang China; ^3^ Department of Gastrointestinal Surgery, Guangxi Medical University Cancer Hospital Guangxi Clinical Research Center for Colorectal Cancer Nanning, Guangxi Autonomous Region China; ^4^ Department of Gastrointestinal Surgery The First Affiliated Hospital of Zhejiang Chinese Medical University Hangzhou China; ^5^ Department of Big Data in Health Science School of Public Health and The Second Affiliated Hospital Zhejiang University School of Medicine Hangzhou Zhejiang China; ^6^ Shandong Provincial Key Laboratory of Animal Cell and Developmental Biology School of Life Sciences Shandong University Qingdao China; ^7^ Institute of Translational Medicine Zhejiang University School of Medicine Hangzhou China; ^8^ State Key Laboratory of Biomembrane and Membrane Biotechnology School of Life Science Tsinghua University Beijing China

**Keywords:** colorectal cancer, deadenylase, patient‐derived tumor cell cluster, PNLDC1, TUBB4B

## Abstract

Colorectal cancer (CRC) remains a leading cause of cancer‐related mortality, highlighting the urgent need for novel therapeutic strategies. In this study, we find that PARN‐like Domain Containing protein‐1 (PNLDC1), an endoplasmic reticulum‐localized deadenylase, lower expresses in 91 clinical CRC tissues. Using both in vitro and in vivo models, we identify PNLDC1 as a key tumor suppressor by modulating cell cycle progression in CRC. RNA‐immunoprecipitation and sequencing analyses reveal that PNLDC1 binds cell cycle‐associated mRNAs, with *TUBB4B* emerging as a critical target. PNLDC1 downregulates *TUBB4B* mRNA, whose regulated degradation is essential for PNLDC1's tumor‐suppressive function. Restoration of TUBB4B expression counteracts PNLDC1‐mediated suppression of CRC cell proliferation, underscoring its pivotal role in CRC pathogenesis. Importantly, we demonstrate that the TUBB4B inhibitor, mebendazole, effectively suppresses the enhanced proliferation in lower‐expression PNLDC1 models, including cell, mouse xenografts, and patient‐derived tumor‐like cell clusters, positioning it as a promising therapeutic agent. These findings establish PNLDC1 as a valuable biomarker and therapeutic target, paving the way for developing novel CRC treatments.

## Graphical abstract

The deficiency of PNLDC1 exacerbates the advancement of CRC. PNLDC1 interacts with *TUBB4B* mRNA to regulate the p53–p21–CDK2–cyclin E2 pathway, thereby influencing the G2/M phase of the cell cycle.

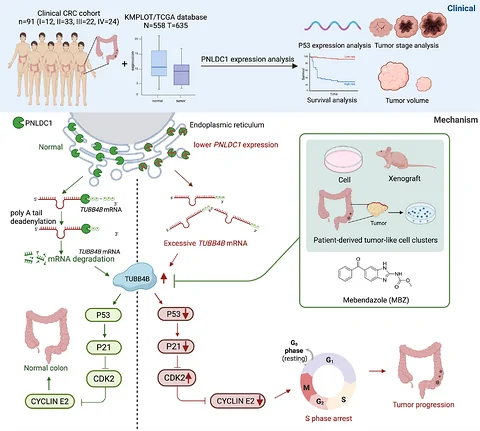

## 1 Main

Colorectal cancer (CRC) is the third most prevalent cancer globally, accounting for approximately 10% of all cancer cases [1]. In 2023 alone, 153,020 individuals were diagnosed with CRC, including 19,550 patients under the age of 50 [2]. Due to its tendency for early‐stage metastasis, CRC is the second leading cause of cancer‐related mortality in high‐income regions and ranks fourth in lower‐income areas [3, 4]. Despite advancements in CRC diagnostics and treatment, the 5‐year survival rate remains suboptimal, around 56.5% in China and Europe and above 64% in United States [5]. CRC significantly impacts patients' quality of life and imposes substantial financial burdens, with annual treatment costs for stage I to stage IV CRC ranging from 56,099 to 82,729 CNY [6, 7]. These challenges highlight the urgent need for further research into CRC mechanisms to improve screening and treatment strategies.

Currently, standard therapeutic targets for CRC include EGFR, VEGF, and immune checkpoint inhibitors, which have shown varied effectiveness across patient subgroups [8, 9]. These therapies focus primarily on cell signaling pathways and tumor angiogenesis but are often limited by resistance mechanisms and adverse effects. The exploration of alternative molecular mechanisms in cancer has opened avenues for identifying new strategies that could supplement existing CRC treatments. Both genetic and environmental factors contribute to CRC etiology [10], and one area of particular interest is the regulation of gene expression through mRNA metabolism [11, 12, 13]. The timely degradation of aberrant mRNAs is essential for maintaining normal cellular function, and disruptions in these processes have been linked to various cancers. However, the full scope of how mRNA decay impacts CRC progression remains incompletely understood.

Deadenylation is a critical process in mRNA regulation that often initiates mRNA decay [14]. Various deadenylases, including CCR4, Nocturnin, PAN, CAF1, PARN, and PARN‐like Domain Containing protein‐1 (PNLDC1), play distinct roles in maintaining mRNA homeostasis [14, 15, 16]. Previous bioinformatic analyses have implicated PNLDC1 in CRC; however, its precise role—whether acting as a proto‐oncogene or tumor suppressor—remains debated [17, 18]. The subcellular distribution of eukaryotic mRNAs can precisely regulate protein expression and interfere with important physiological processes [19, 20]. Unlike other well‐characterized deadenylases within the cytoplasm and nuclear/mitochondrial matrix [21, 22], the function of PNLDC1 is undiscovered. Interestingly, PNLDC1 stands out as the only deadenylase potentially localized in the endoplasmic reticulum (ER) [23], and where mRNAs are enriched under physiological conditions [24, 25, 26]. Hence, understanding whether ER‐located PNLDC1's function in CRC could shed light on novel therapeutic mechanisms.

The tubulin beta 4B class IVb (TUBB4B) protein belongs to the β‐tubulin family and is an essential gene that regulates the cell cycle. Moreover, TUBB4B was reported to be involved in cancer. The epithelial–mesenchymal transition (EMT) process is dependent on TUBB4B decrease in CRC [27]. Overexpression of TUBB4B suppressed the EMT process of lung cancer cells [28, 29]. Inhibition of AGTPBP1 lead to TUBB4B downregulation and arrest G2/M cell cycle phase in pancreatic cancer [30].

In this study, we identify PNLDC1, an ER‐localized deadenylase, as a tumor suppressor in CRC using in vivo and in vitro models. Through RNA‐immunoprecipitation (RIP) and subsequent sequencing, we demonstrate that PNLDC1 binds a series of cell cycle‐related genes, highlighting *TUBB4B* as a key target. Our findings elucidate the underlying molecular mechanisms of PNLDC1's action, offering insight into how disruptions in mRNA decay contribute to CRC. Furthermore, we explore the therapeutic potential of the TUBB4B inhibitor mebendazole (MBZ), proposing it as a promising new strategy for CRC treatment.

## 2 Results

### 2.1 *PNLDC1* Is Downregulated in CRC Tissues

To investigate the potential role of PNLDC1 in CRC, we used our own clinical cohort specimens as well as public databases (Figure 1A). We initially analyzed its expression using publicly available online databases, including KMplot (http://kmplot.com/analysis/index.php?p = service), GEPIA (http://gepia2.cancer‐pku.cn/#survival), and cBio (http://www.cbioportal.org) which revealed significantly lower *PNLDC1* mRNA levels in CRC tissues compared to normal tissues (Figure 1B and Figure S1A). These results collectively indicate that *PNLDC1* exhibits low expression levels in tumor tissues. Tumor mutation burden (TMB) score was also positively correlated with *PNLDC1* methylation level (Figure S1B). *PNLDC1* expression demonstrated no significant association with tumor metastasis (Figure S1C). To validate the relationship between PNLDC1 and tumor based on cBio Portal, we examined PNLDC1 expression in 91 paired CRC and adjacent non‐cancerous tissues from clinical samples (Table S4). *PNLDC1* mRNA expression was significantly downregulated in CRC tissues compared to normal tissues (Figure 1C). Immunohistochemical (IHC) staining further demonstrated decreased PNLDC1 levels in CRC tissues (Figure 1D). RT‐qPCR and Western blot (WB) analyses (10 µg protein extract was loaded into each lane) demonstrated consistently reduced *PNLDC1* mRNA and protein levels in tumor tissues compared with paracancer tissues (Figure 1E,F). Notably, patients with *TP53* mutations exhibited significantly lower PNLDC1 expression, whereas no significant difference was observed in patients with *BRAF* mutations (Figure 1G,H). Additionally, no correlation was identified between PNLDC1 expression levels and overall survival, tumor stage, position, volume, or pathological classification (Figure S1D–I). Collectively, these findings suggest that PNLDC1 may serve as a diagnostic marker for CRC.

**FIGURE 1:**
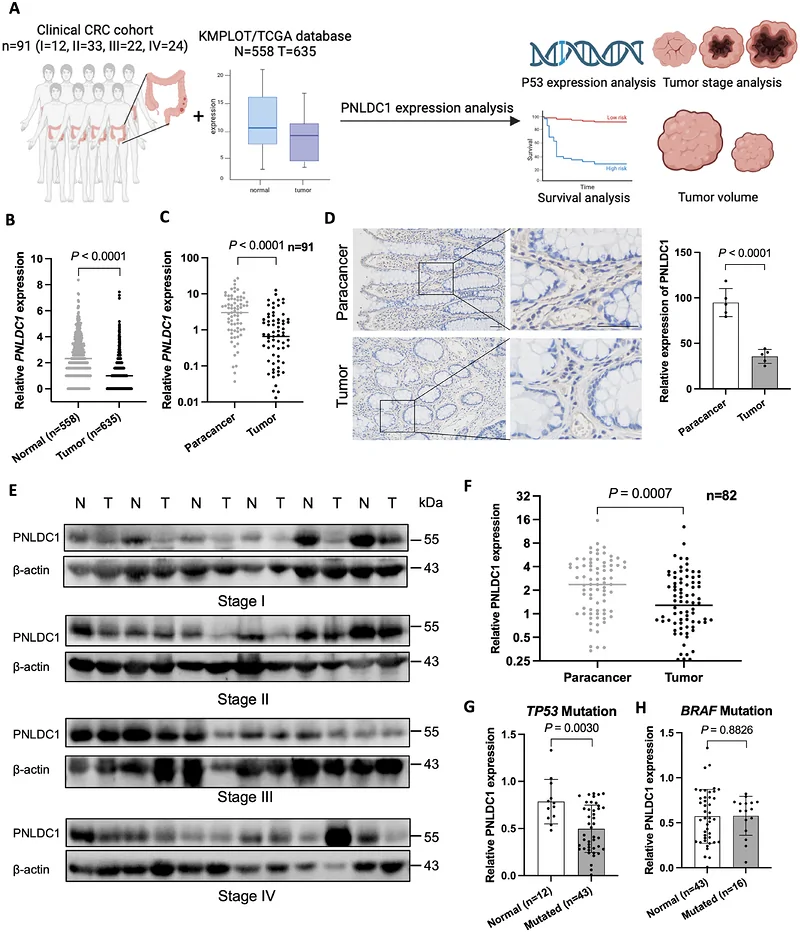
Analysis of PNLDC1 expression in CRC patients. (A) Schematic overview of the strategy used for analyzing PNLDC1 expression in CRC patients. (B) Comparison of *PNLDC1* mRNA expression between cancerous (*n* = 635) and normal colorectal tissues (*n* = 558) based on the KMplot database, showing lower *PNLDC1* levels in cancer tissues. (C) RT‐qPCR analysis of *PNLDC1* mRNA expression in CRC patient samples, highlighting reduced levels in tumor tissues compared to adjacent non‐tumor tissues (*n* = 91). (D) IHC analysis of PNLDC1 expression in CRC tissues, with representative images shown at a scale of 50 µm (*n* = 5). (E) Western blot analysis showing PNLDC1 expression across different CRC stages, comparing normal (N) and tumor (T) tissues. (F) Quantitative analysis of the WB results from panel (E) (*n* = 82). (G) Comparative analysis of PNLDC1 expression in CRC patient tissues with *TP53* mutations (*n* = 43) versus non‐mutated tissues (*n* = 12). (H) Comparative analysis of PNLDC1 expression in CRC patient tissues with *BRAF* mutations (*n* = 16) versus non‐mutated (*n* = 43) tissues.

### 2.2 Identification of *PNLDC1* as a Suppressor in CRC

To further explore the role of PNLDC1 in CRC, the expression of PNLDC1 was assessed in various CRC cell lines. HCT116 cells, which exhibited high PNLDC1 expression, were selected for *PNLDC1* knockout (KO) experiments (Figure S2A–C). Two independent isogenic *PNLDC1*‐KO clones named C10 and A5 were generated (Figure S2C). Colony formation and CCK‐8 assays showed that *PNLDC1* KO enhanced cell proliferation and colony‐forming ability (Figure 2A,B and Figure S2E–F). Then siRNA targeting *PNLDC1* was performed to knockdown (KD) *PNLDC1* which was verified by WB in HCT116 cells (Figure S2D). CCK‐8 assay revealed that KD of *PNLDC1* led to significant increase of cell viability (Figure S2H). Additionally, wound healing and transwell assays demonstrated that *PNLDC1* KO promoted cell migration and invasion (Figure 2C,G–H and Figure S2H). Cell cycle detection via flow cytometry and WB revealed an increased percentage of S phase cells and reduced G2/M phase cells in *PNLDC1* KO cells (Figure 2I–J). Therefore, we co‐stained these synchronized and arrested cells using antibodies against Phospho‐Histone H3 (Ser10) (p‐Histone H3), a well‐established marker specifically labeling chromatin in cells undergoing mitosis, and α‐tubulin, to visualize the microtubule cytoskeleton and spindle morphology. This detailed assessment revealed that *PNLDC1*‐KO cells exhibited a reduced proportion of cells in M‐phase (as quantified by pH3 positivity) compared to control cells following release from nocodazole. However, careful examination of mitotic cell morphology did not reveal significant abnormalities (Figure S2G). In addition, decreased p53 and p21 levels and increased cyclin E2 and CDK2 levels were observed (Figure 2K). To complement these findings, stable *PNLDC1*‐overexpressing (OE) HCT116 and SW480 cell lines were established (Figure S2 I,K‐L). OE of *PNLDC1* resulted in reduced cell viability and migration (Figure 2D–F and Figure S2J,M–N). In addition, the accumulation of the cell cycle inhibitor p21 in *PNLDC1* OE cell lines suggested that PNLDC1 induces cell cycle arrest (Figure 2K).

**FIGURE 2:**
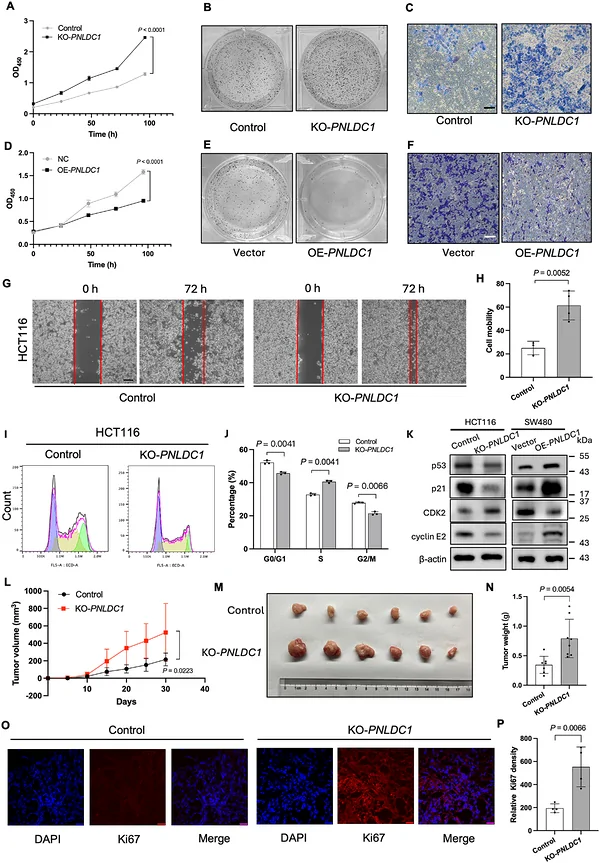
*PNLDC1* KO enhances the malignant phenotype of CRC cells. (A, B) *PNLDC1* KO in HCT116 cells increased cell proliferation as demonstrated by CCK‐8 assays and colony formation assays (*n* = 3). (C) Transwell migration assays showing enhanced migration in KO*‐PNLDC1* HCT116 cells compared to control cells (scale: 100 µm). (D, E) OE of *PNLDC1* in HCT116 cells inhibited cell proliferation as shown by CCK‐8 assays and colony formation assays (*n* = 3). (F) Transwell migration assays demonstrating reduced migration in *PNLDC1*‐OE HCT116 cells compared to vector controls (scale: 100 µm). (G, H) Scratch assays indicating that *PNLDC1* KO promotes cell migration in HCT116 cells (*n* = 3, scale: 100 µm). (I, J) Cell cycle analysis showing changes in cell cycle distribution in KO*‐PNLDC1* HCT116 cells (*n* = 3). (K) WB analysis of p53, p21, CDK2, and cyclin E2 protein levels in control and KO*‐PNLDC1* HCT116 cells. (L) Tumor growth curve over time in mice implanted with control or KO*‐PNLDC1* HCT116 cells (*n* = 6 mice per group). (M) Images of tumors excised from subcutaneous xenograft models treated with HCT116 control and KO*‐PNLDC1* cells (*n* = 6 mice per group). (N) Tumor weight comparison between the control and KO*‐PNLDC1* groups (*n* = 6 mice per group). (O, P) Immunofluorescence analysis of Ki67 expression in xenograft tumors, indicating higher proliferation in KO*‐PNLDC1* tumors (*n* = 4, scale: 20 µm).

In vivo experiments further validated these observations. NOD/ShiLtJGpt‐Prkdcem26Cd52II2rgem26Cd22/Nju (NCG) mice injected with *PNLDC1* KO HCT116 cells exhibited tumors that grew approximately twice as fast as those in the control group (Figure 2L,M). After 30 days, tumor weights were higher in the *PNLDC1* KO group, with increased Ki67 expression observed (Figure 2N–P). These results underscore PNLDC1 acts as a tumor suppressor in CRC both in vitro and in vivo.

### 2.3 Subcellular Localization of PNLDC1

To further elucidate the mechanism of PNLDC1 in CRC, we first investigated the subcellular localization of endogenous PNLDC1 in cell lines. Using RT‐qPCR, we identified HCT116 and MRC5 cells with high PNLDC1 expression for further analysis (Figure S3A). Immunofluorescence staining revealed that PNLDC1 exhibited a strong co‐localization with the PDI, ER marker, indicated by higher Pearson's correlation coefficients compared to other ER markers, calnexin and BiP (Figure 3A and Figure S3B). Similarly, cell fractionation and WB analysis further confirmed the enrichment of PNLDC1 in membrane fractions (Figure 3B). GFP‐tagged exogenous PNLDC1 was also detected in HEK293T cells, consistent with previous findings that exogenous PNLDC1 localized to the ER (Figure S3C) [24]. These findings demonstrate that PNLDC1 is predominantly localized in the ER.

**FIGURE 3:**
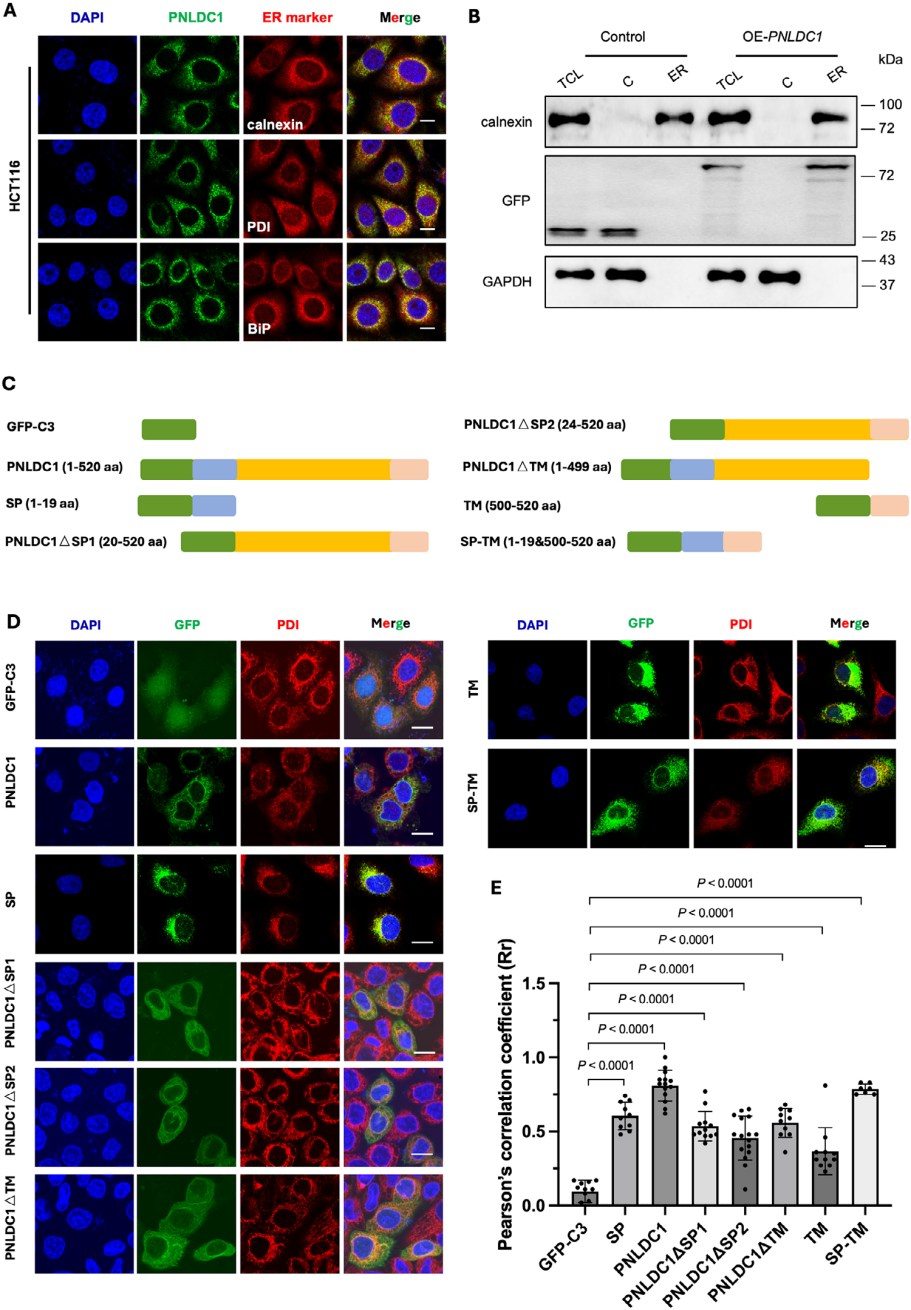
Domain mapping of PNLDC1 localization to the ER. (A) Representative fluorescence confocal images showing the localization of endogenous PNLDC1 in HCT116 cells, which express high levels of PNLDC1. ER markers calnexin, BiP, and PDI were used for co‐localization analysis (scale: 10 µm). (B) WB analysis demonstrating the ER localization of PNLDC1 in HEK293T cells. Total cell lysates (TCL), cytosolic (C), and ER‐enriched (ER) fractions were analyzed. (C) Schematic representation of full‐length PNLDC1 and various truncated constructs, including SP (1‐19 aa), PNLDC1ΔSP1 (20‐520 aa), PNLDC1ΔSP2 (24‐520 aa), PNLDC1ΔTM (1‐499 aa), TM (500‐520 aa), and SP‐TM (1‐19 and 500–520 aa). (D) Representative fluorescence confocal images showing the subcellular localization of truncated PNLDC1 constructs in HEK293T cells (scale: 10 µm).

Bioinformatics modeling predicted the isoform (520 aa) harbors a signal peptide (SP) and a transmembrane (TM) region. Immunofluorescence of PNLDC1 truncations revealed that both the SP and TM domains were necessary for ER localization (Figure 3C–E), suggesting a structural basis for its role in ER.

### 2.4 Identification of PNLDC1‐Binding Motifs on RNA

To explore how ER‐localized PNLDC1 contributes to CRC progression, we assessed its enzymatic activity. Deadenylation assays performed on cell lysates and ER‐enriched fractions of HA‐PNLDC1‐expressing HEK293T cells demonstrated significantly higher AMP production (peak at 18.8 nm) compared to vector controls (Figure 4A and Figure S4A). As a product of deadenylation, AMP reflects the cleavage of poly(A) tails from mRNA, confirming PNLDC1's robust deadenylase activity. RIP sequencing (RIP‐seq) was conducted to identify PNLDC1‐binding targets in HCT116 and HEK293T cells (Figure 4B,C). RIP peak distribution analysis demonstrated PNLDC1 was predominantly mapped to 3′UTR of mRNAs, with a preference for adenine‐rich motifs in both cell lines (Figure 4D,E), consistent with previous studies showing PNLDC1 has poly(A) tail affinity [31]. Gene ontology (GO) and Gene Set Enrichment Analysis (GSEA) indicated that these target mRNAs are heavily involved in cell cycle regulation, further linking PNLDC1's deadenylase activity to CRC pathogenesis (Figure 4F–G and Figure S4B,C). RT‐qPCR analysis in CRC patients’ tissues identified *TUBB4B* as a key RNA target of PNLDC1 (Figure 4H and Figure S4D), validated by DNA electrophoresis (Figure 4I). To confirm PNLDC1's role in regulating *TUBB4B* mRNA stability, actinomycin D treatment was performed, revealing significantly delayed mRNA degradation in *PNLDC1* KO cells compared to controls (Figure 4J). To determine the functional domains of PNLDC1 involved in mRNA binding, we identified 15–490 amino acids (aa) region as the mRNA binding domain based on InterPro annotations (https://ebi.ac.uk). Corresponding truncation plasmids were constructed (Figure 4K). Moreover, we discovered that the 15–490 aa region is responsible for mRNA binding by using hybridization chain reaction (HCR) assays (Figure 4L). Since PNLDC1 is an ER‐localized protein, to investigate how its aberrant expression modulates ER stress pathways, we analyzed the expression of key pathway components (Figure S4E). Our results demonstrate that PNLDC1 selectively regulates the ATF4–CHOP axis of ER stress pathway by binding to specific regions of *ATF4* mRNA and promoting its destabilization (Figure S4E–I).

**FIGURE 4:**
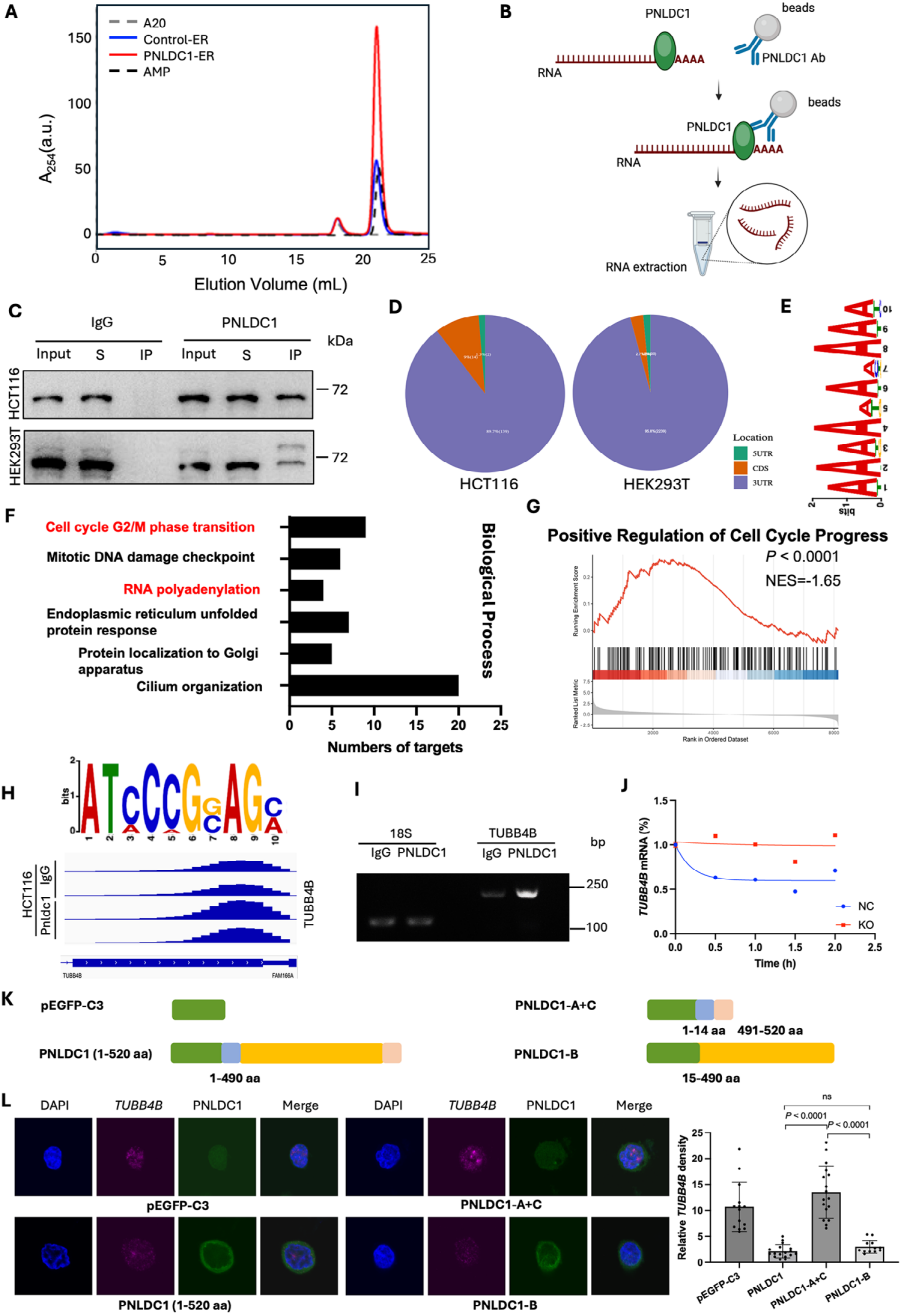
Identification of genome‐wide PNLDC1‐binding sites on RNA. (A) Deadenylase activity assay of microsome fractions isolated from HEK293T cells using size‐exclusion chromatography (SEC). Total protein content in each fraction was standardized based on bovine serum albumin (BSA) quantification. (B) Schematic representation of the RIP workflow used for identifying PNLDC1‐binding sites. (C) RIP analysis of PNLDC1 in HCT116 and HEK293T cells. (D) Distribution profile of PNLDC1‐binding peaks across the genome. (E) Analysis showing PNLDC1's poly(A)‐tail specificity. (F) GSEA indicating involvement in the regulation of the ER unfolded protein response and negative regulation of the response to ER stress. (G) Gene ontology (GO) analysis of the immunoprecipitated transcriptome, highlighting relevant biological processes. (H) Enriched sequence motifs identified in the top PNLDC1‐binding sites and *TUBB4B* as a representative target. (I) Electrophoresis results of *TUBB4B* RT‐qPCR products following RIP, confirming PNLDC1 binding. (J) Decay kinetics of *TUBB4B* mRNA measured by quantitative RT‐qPCR, showing reduced degradation rates under *PNLDC1*‐KO. (K) Schematic diagram of truncation plasmid construction. (L) HCR analysis shows the expression of *TUBB4B* mRNA levels of each truncation and control.

### 2.5 PNLDC1 Regulated the Expression of TUBB4B

To investigate the regulatory relationship between PNLDC1 and TUBB4B, we measured TUBB4B expression in online database, cell lines, mice xenografts, and patient tissues.

We conducted comprehensive analyses across three authoritative portals (TIMER2.0 http://timer.comp‐genomics.org, cBioPortal http://www.cbioportal.org, and LinkedOmics https://www.linkedomics.org) on the correlation between *PNLDC1* and *TUBB4B* and found that the expression of *PNLDC1* and *TUBB4B* is inversely correlated (Figure S5A–C).

**FIGURE 5:**
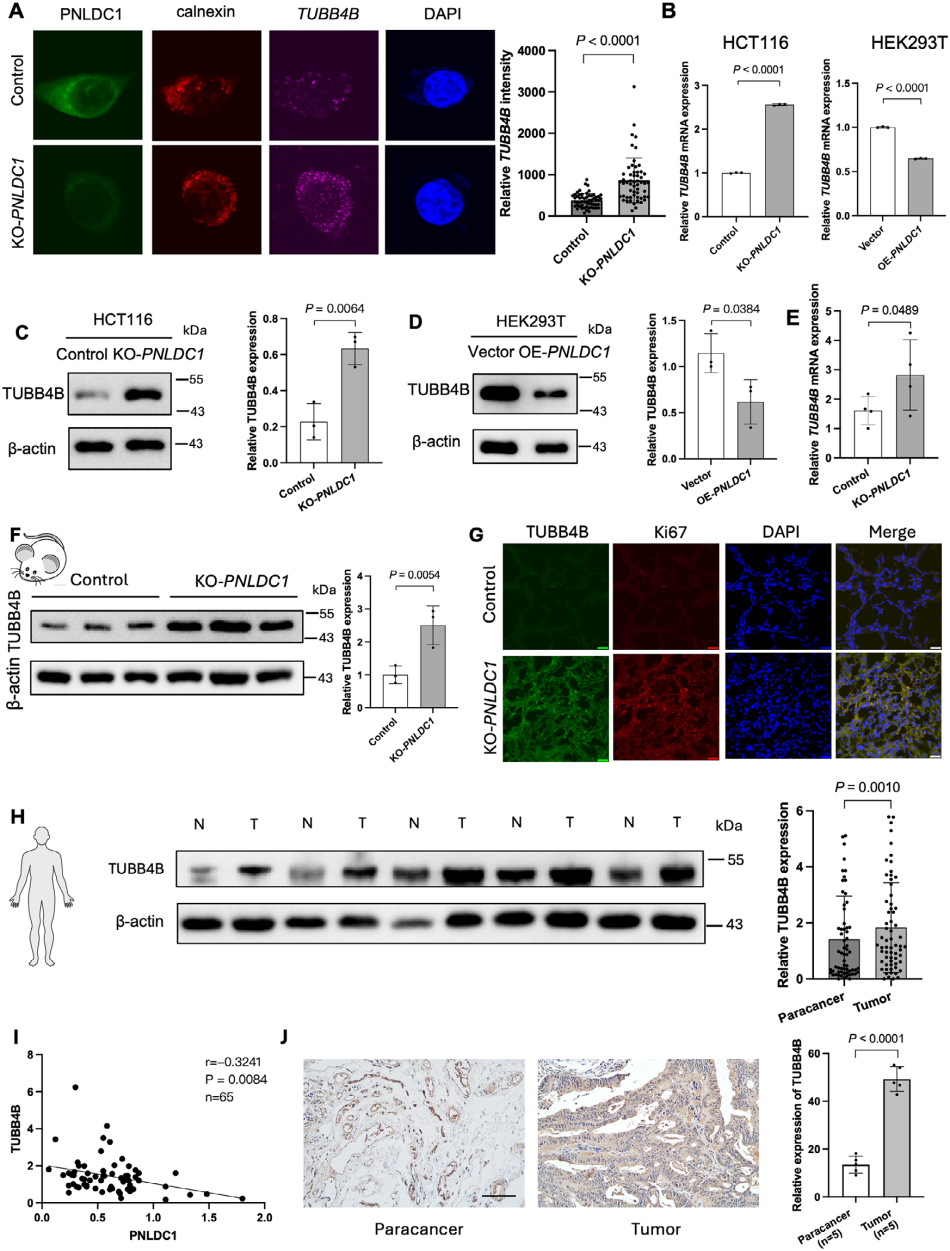
*PNLDC1* KO stabilizes the mRNA expression of *TUBB4B*. (A) HCR analysis shows increased *TUBB4B* mRNA levels in KO*‐PNLDC1* HCT116 cells compared to controls (*n* = 56). (B) RT‐qPCR results indicating higher *TUBB4B* mRNA levels in KO*‐PNLDC1* HCT116 cells and reduced levels in OE*‐PNLDC1* HEK293T cells compared to controls. (C) WB analysis of TUBB4B protein expression in HCT116 cells. (D) WB analysis of TUBB4B protein expression in HEK293T cells. (E) RT‐qPCR results confirming *TUBB4B* mRNA expression in control and KO*‐PNLDC1* HCT116 cells. (F) WB analysis showing TUBB4B protein expression in tumors from xenograft mice treated with control and KO*‐PNLDC1* HCT116 cells (*n* = 3). (G) Immunofluorescence images of TUBB4B and Ki67 expression in xenograft tumors treated with HCT116 control and KO*‐PNLDC1* cells (scale: 20 µm). (H) WB analysis comparing TUBB4B expression in paracancerous and cancerous tissues (*n* = 65). (I) Correlation analysis of PNLDC1 and TUBB4B expression in CRC patient samples, showing an inverse relationship (*n* = 65). (J) IHC of TUBB4B of paracancer and tumor tissues from CRC patients (*n* = 5, scale: 100 µm).

HCR assays indicated that *TUBB4B* mRNA levels were elevated in *PNLDC1* KO cells and reduced in *PNLDC1* OE cells (Figure 5A and Figure S5D). WB analysis confirmed that TUBB4B protein levels were upregulated in *PNLDC1* KO and downregulated in *PNLDC1* OE cells (Figure 5B–E). In mouse xenograft models, TUBB4B expression was notably higher in tumors derived from *PNLDC1* KO cells (Figure 5F), consistent with TUBB4B upregulation in tumor tissues compared to paracancerous tissues in patients (Figure 5G). Importantly, clinical correlation analysis revealed an inverse relationship between PNLDC1 and TUBB4B expression (Figure 5H–J and Figure S5E). These findings establish *TUBB4B* as a critical downstream target of PNLDC1.

### 2.6 Targeting TUBB4B Reverses PNLDC1‐Mediated Cell Cycle Dysregulation

To further investigate the impact of PNLDC1 on the cell cycle, we constructed a protein–protein interaction (PPI) network using the STRING database, which revealed a direct interaction between TUBB4B and p53 (Figure S6A). To verify the result of STRING database, co‐immunoprecipitation (Co‐IP) was performed in CRC tissue and found that TUBB4B and p53 co‐immunoprecipitated, indicating their protein interaction (Figure 6A). siRNA‐mediated KD of *TUBB4B* in *PNLDC1* KO HCT116 cells restored p53 and p21 levels without affecting CDK2 and cyclin E2 (Figure 6B and Table S3). From Drug–Gene Interaction Database (DGIdb), we identified 84 known TUBB4B inhibitors. Among all candidates, MBZ demonstrated a higher binding affinity score (0.12), justifying its prioritization for pharmacological effects. Pharmacological inhibition of TUBB4B using MBZ further validated these findings. MBZ treatment suppressed cell growth in a dose‐dependent manner (IC_50_ = 0.53 µM; Figure 6C,D and Figure S6B) and induced G2/M phase arrest (Figure 6E and Figure S6D). In vivo, MBZ treatment (50 mg kg^−1^, oral administration) significantly inhibited tumor growth in *PNLDC1* KO cell‐derived xenografts (CDX) (Figure 6F–I and Figure S6C), with reduced TUBB4B expression (Figure 6J,K and Figure S6E). Furthermore, we detected the expression of tubulins after 96‐h MBZ treatment, and found that though *PNLDC1* KD did not affect tubulins’ expression, the administration of MBZ does not induce compensatory upregulation of other microtubule proteins. It causes a decrease in α‐tubulin and β‐tubulin expression but does not alter the levels of γ‐tubulin or the β‐tubulin subtype TUBB3 (Figure S6F–H). Moreover, the combinatorial regimen of MBZ and cisplatin was evaluated, and observed a synergistic effect between the two agents (Figure S6I–J). These findings highlight the therapeutic potential of targeting the PNLDC1‐TUBB4B axis in CRC, demonstrating that inhibiting TUBB4B effectively reverses the dysregulated cell cycle and tumor growth driven by *PNLDC1* deficiency.

**FIGURE 6:**
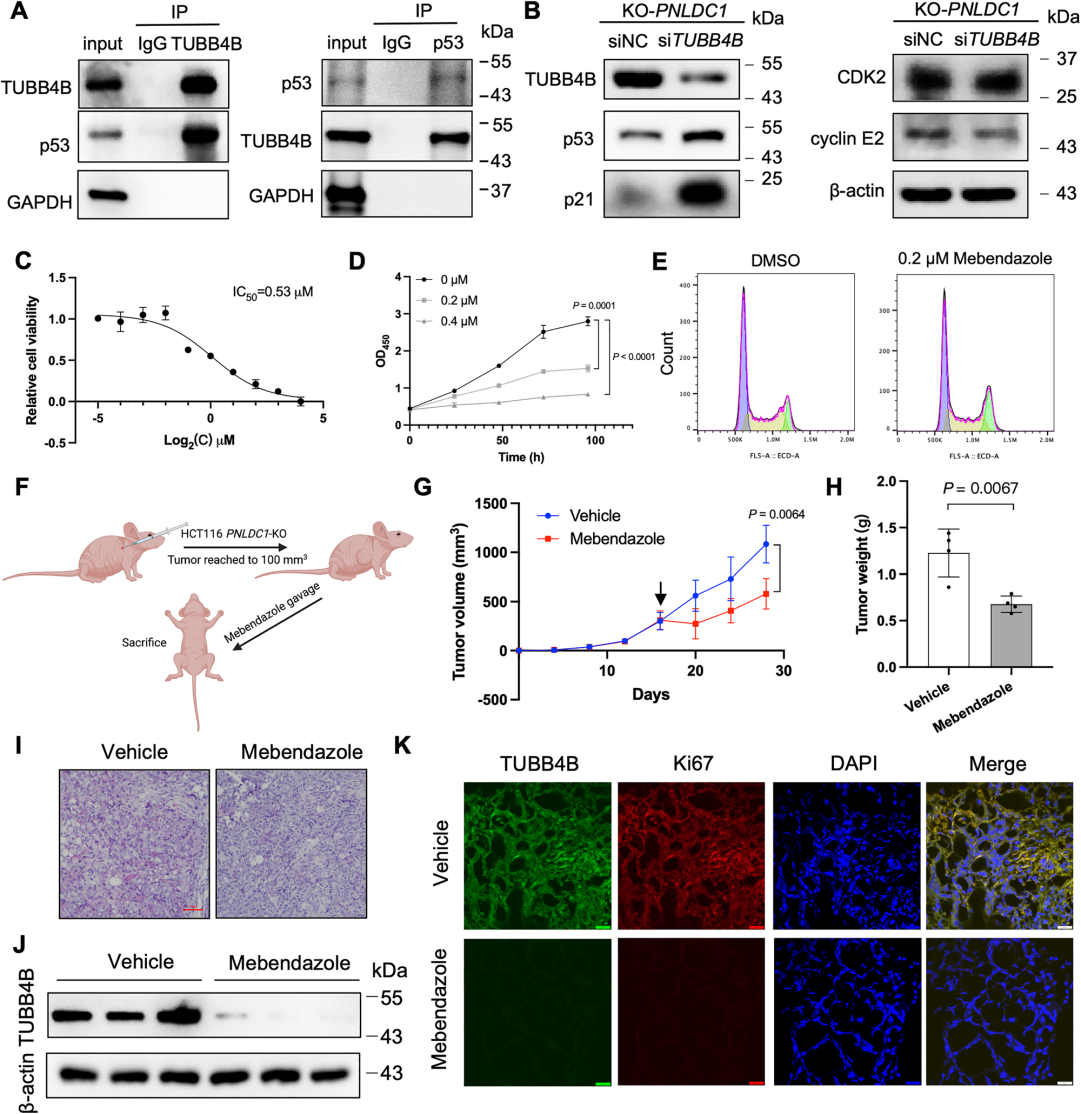
Inhibition of TUBB4B reverses tumor progression caused by *PNLDC1* deficiency. (A) TUBB4B‐p53 interaction validated by Co‐IP in CRC tissue. (B) WB analysis showing the expression levels of TUBB4B, p53, p21, CDK2, and cyclin E2 in KO*‐PNLDC1* HCT116 cells and those treated with si*TUBB4B* (*n* = 3). (C) Dose–response curves of HCT116 cells treated with MBZ for 48 h (*n* = 3). (D) CCK‐8 assay results indicating that MBZ inhibits HCT116 cell growth in a dose‐ and time‐dependent manner (*n* = 3). (E) Analysis showing that MBZ treatment increases the proportion of cells in the G2/M phase (*n* = 3). (F) Schematic representation of the MBZ administration strategy used in xenograft models. (G, H) Tumor growth curves and tumor weights of HCT116*‐PNLDC1*‐KO xenografts treated with vehicle or MBZ via oral gavage (arrow shows the time of MBZ administration) (*n* = 4). (I) Hematoxylin and eosin (H&E) staining of xenograft tumors treated with vehicle or MBZ (scale: 50 µm). (J) WB analysis of TUBB4B protein expression in xenograft tumors derived from KO*‐PNLDC1* HCT116 cells treated with vehicle or MBZ (*n* = 3). (K) Immunofluorescence images of xenograft tumors showing TUBB4B expression in KO*‐PNLDC1* HCT116 cells treated with vehicle or MBZ (scale: 20 µm).

### 2.7 Therapeutic Potential of MBZ in Patient‐Derived Tumor‐Like Cell Clusters

To better simulate clinical conditions, we evaluated the effect of TUBB4B inhibition using MBZ in patient‐derived tumor‐like cell clusters (PTCs) (Figure 7A–E) [32]. MBZ treatment resulted in a significant, concentration‐dependent reduction in PTC viability (Figure 7D and Figure S7A). Additionally, measurements of cell area revealed that MBZ treatment effectively decreased PTC size (Figure 7F and Figure S7B–D). These results underscore the potential of MBZ as a therapeutic agent targeting the PNLDC1‐TUBB4B axis in CRC, further supporting its clinical applicability in reducing tumor cell viability and growth.

**FIGURE 7:**
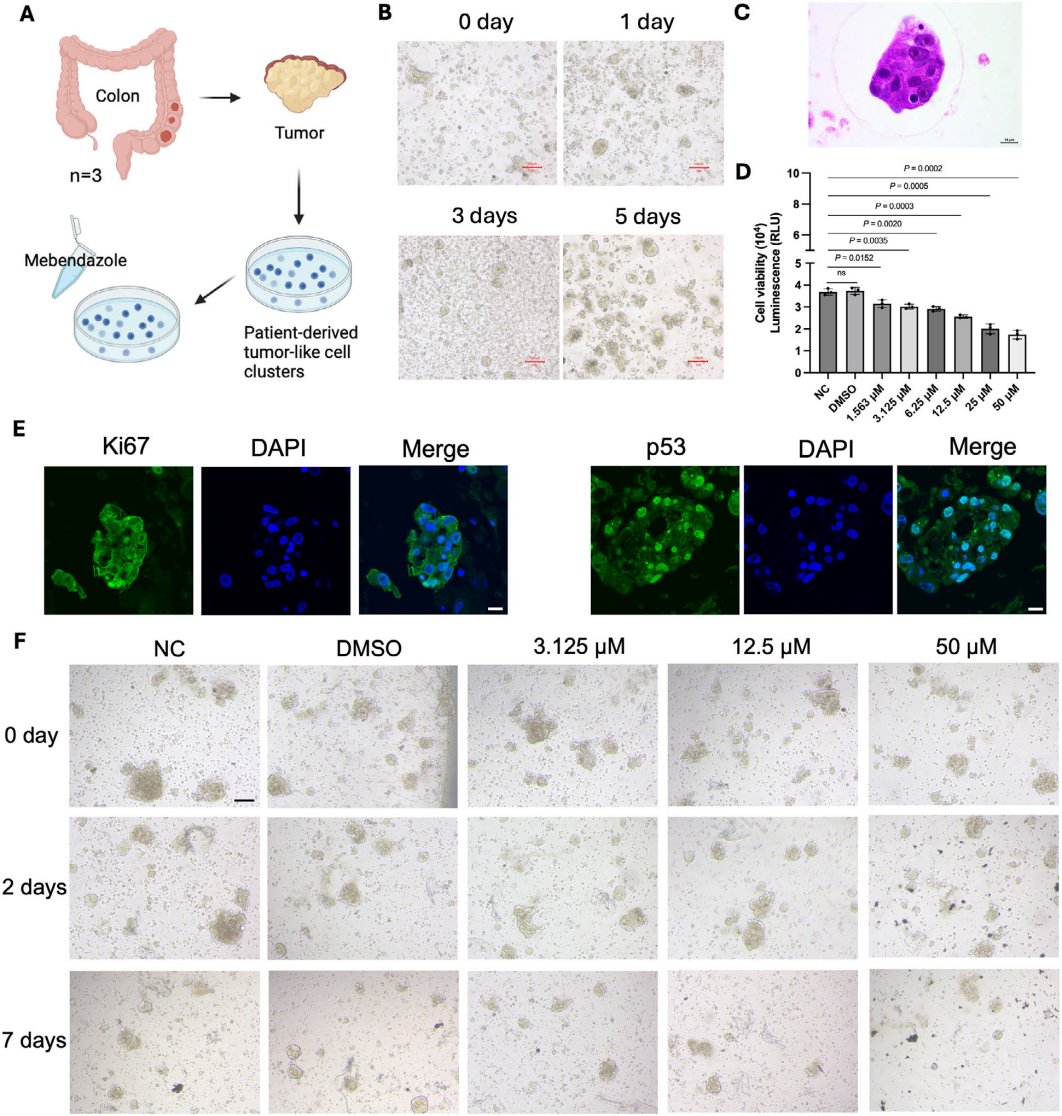
The effect of MBZ on PTCs. (A) Schematic overview of the process for generating PTCs. (B) Representative images showing the successful formation of PTCs (scale: 100 µm). (C) Hematoxylin and eosin (H&E) staining of PTCs, confirming tissue morphology (scale: 10 µm). (D) Analysis demonstrating that high concentrations of MBZ significantly reduce PTC cell viability (*n* = 3). (E) Immunofluorescence staining of Ki67 and p53 in PTCs, indicating the effect of MBZ on proliferation and tumor suppression markers (scale: 10 µm). (F) Dose‐dependent reduction in PTC area following MBZ treatment (scale: 100 µm).

## 3 Discussion

PNLDC1 is an RNA‐binding protein that plays a well‐established role in spermatogenesis; however, recent evidence also links it to the pathogenesis of various cancers, including ovarian cancer and CRC by bioinformatic analyses [18, 33]. Its function as either a promoter or suppressor of tumorigenesis has remained unresolved. In this study, we establish PNLDC1 as a novel ER‐localized deadenylase with tumor‐suppressive properties in CRC. By mediating the degradation of *TUBB4B* mRNA, PNLDC1 modulates cell cycle progression. Cancer‐testis antigens (CTAs) are characterized by highly restricted expression in germline tissues and aberrant activation in diverse malignancies [34]. The striking downregulation of PNLDC1 in CRC together with its testis‐restricted expression pattern strongly suggests that PNLDC1 exhibits CTA‐like expression features.

mRNA degradation plays a fundamental role in post‑transcriptional gene regulation and is essential for maintaining cellular homeostasis and proteostasis [35]. Deadenylases constitute a major class of enzymes responsible for initiating mRNA decay by removing poly(A) tails, thereby modulating transcript stability, gene expression, and key cellular processes including cell cycle progression and apoptosis [36, 37, 38, 39, 40]. Dysregulation of deadenylation has been increasingly implicated in cancer, as exemplified by PARN, CNOT6, CNOT7, and NOCT, which affect tumor growth and metabolism by stabilizing oncogenic transcripts or destabilizing tumor suppressor mRNAs [41]. Our previous work and others have shown that CCR4 and PARN regulate proliferation and tumorigenicity by modulating p21 expression [37, 42]. Similarly, blocking CCR4 suppressed *SLC7A11* mRNA deadenylation in hepatoblastoma [43]. Collectively, these studies underline the central role of deadenylases in cancer development.

Accumulating evidence suggests that aberrant mRNA stability serves as a master regulatory mechanism in CRC carcinogenesis. Stabilization of oncogenes such as *MYC*, *CCND1*, and *VEGFA* enhances proliferative and angiogenic signaling, while enhanced decay of tumor suppressors including *PTEN* and *CDKN1A* disrupts growth control [44, 45]. In addition, stabilization of *CTNNB1* amplifies Wnt/β‑catenin signaling [46], altered 3′UTR regulation of *EGFR* modulates receptor abundance [47], and *TP53* mRNA instability compromises DNA damage responses [48]. These observations underscore that malignant phenotypes—ranging from proliferation to EMT, metastasis and therapy resistance—can emerge from perturbations in mRNA decay pathways [49, 50].

In the context of CRC, certain deadenylases have emerged as modulators of tumor progression. For example, inhibition of CNOT2, a subunit of the CCR4–NOT complex, has been shown to activate p53 signaling and induce apoptosis in CRC cells [51]. Although PARN has been implicated in mRNA decay and the DNA damage response in cancers such as esophageal [52], glioblastoma [53], gastric, and breast cancers [42, 54], there is currently no direct evidence linking PARN dysfunction to CRC. Similarly, NOCT, a circadian‐regulated deadenylase within the CCR4 family, has demonstrated metabolic functions, but its role in CRC remains underexplored [55]. However, most studies to date have focused on cytoplasmic deadenylation, leaving the spatial organization of mRNA decay within subcellular compartments largely unexplored in CRC.

To our knowledge, PNLDC1 is the only ER‐localized deadenylase, distinct from canonical CCR4–NOT members. Previously reported for its role in spermatogenesis through piRNA trimming and transposon silencing [56, 57], its function in mRNA regulation has not been characterized. Our study is the first to confirm the specific domains responsible for PNLDC1's ER localization, demonstrating that the SP and TM regions are essential for its targeting (Figure 3). Here, we demonstrate that PNLDC1 directly binds *TUBB4B* mRNA through a specific motif (Figure 4), regulating its stability and thereby influencing CRC tumorigenesis. This ER‐localized activity of PNLDC1 represents a novel layer of post‐transcriptional regulation, integrating mRNA decay with cellular stress responses and cell cycle control.

Notably, we observed that *TP53*‑mutant CRC samples displayed reduced *PNLDC1* expression. Although the regulatory mechanism linking *TP53* and PNLDC1 remains undefined, several hypotheses merit further investigation. Wild‐type p53 may transcriptionally activate PNLDC1 via direct promoter binding [58], a function potentially lost or antagonized by mutant p53. Additionally, p53‐regulated microRNAs, such as the miR‐34 family and miR‐17‐92 cluster, may modulate RNA‐binding proteins or deadenylases, thereby influencing PNLDC1 expression indirectly [59, 60, 61]. Future studies should explore whether PNLDC1 harbors p53 response elements within its promoter region or is repressed through p53‐dependent miRNA networks in CRC.

TUBB4B, here identified as a key PNLDC1 target, is known to be enriched in membrane fractions and to play roles in cell cycle regulation [62]. Previous studies showed that TUBB4B downregulation induces G2/M phase arrest in non‐alcoholic fatty liver disease‐associated hepatocellular carcinoma (NAFLD‐HCC) [63]. Additionally, TUBB4B was confirmed to interact with p53 by using Co‐IP assays [64]. Moreover, we observed that PNLDC1 expression was significantly reduced in *TP53*‐mutant CRC samples, but not in those with *BRAF* mutations (Figure 1). Our results suggest that PNLDC1‐mediated degradation of *TUBB4B* mRNA impacted the p53–p21–CDK2–cyclin E2 pathway (Figures 2 and 6), reinforcing PNLDC1's tumor‐suppressive function in CRC [65, 66]. This connection was substantiated by the restoration of p53 and p21 levels when *TUBB4B* was silenced in *PNLDC1* KO cells, confirming the relationship between TUBB4B and p53 signaling in CRC (Figure 6).

MBZ, an FDA‐approved drug known for its inhibitory effect on TUBB4B, was found to reduce CRC cell proliferation and induce G2/M phase arrest in *PNLDC1* KO models. Furthermore, MBZ treatment effectively suppressed the growth of PTCs, positioning it as a promising candidate for CRC therapy targeting the PNLDC1‐TUBB4B axis [67, 68, 69]. These findings pave the way for future research into TUBB4B inhibitors as potential therapeutic agents, potentially in combination with current treatment regimens to enhance CRC patient outcomes. Preclinical reports in glioblastoma [70] and CRC [71] show sustained MBZ efficacy without resistance development. The ongoing Phase 3 trial (NCT03925662) monitoring resistance in metastatic colon cancer patients will provide further clinical validation [72]. Beyond its anti‐cancer potential, MBZ also exerts well‐established antiparasitic effects via partially overlapping mechanisms—namely, inhibition of microtubule polymerization by binding β‐tubulin subunits and blockade of glucose uptake in parasites [73, 74]. Notably, MBZ differs mechanistically from other microtubule‐targeting drugs such as paclitaxel, which stabilizes rather than destabilizes microtubules. This divergence raises the possibility that MBZ may act on TUBB4B with distinct specificity, meriting further mechanistic studies to clarify its precise molecular interactions in CRC and to distinguish its anti‐cancer actions from its antiparasitic properties.

Despite these advances, several limitations should be acknowledged. First, although PNLDC1 downregulation is a key event in CRC, its upstream regulatory mechanisms remain unclear. Future studies should explore transcriptional, epigenetic, or proteasomal control of PNLDC1 expression. Second, our in vivo validation was limited due to ethical constraints; long‐term models or patient‐derived organoids (PDOs) will be essential to assess therapeutic relevance and tumor heterogeneity. Third, while MBZ was identified as a promising TUBB4B inhibitor based on in silico interaction scoring, direct biochemical validation of MBZ–TUBB4B binding will be required in future studies. Additionally, although MBZ engages TUBB4B, its target specificity cannot be determined, and off‑target effects on other tubulin isoforms cannot be excluded. Moreover, while we identified *TUBB4B* as a major target of PNLDC1, the residual CDK2/cyclin E2 levels suggest additional downstream effectors may exist and warrant transcriptome‐wide mRNA stability profiling. Finally, given PNLDC1's ER localization and link to ATF4–CHOP, its role in ER stress response deserves further investigation as a potential node connecting mRNA decay to proteostasis.

In summary, this study identifies PNLDC1 as a key ER‐associated deadenylase with tumor‐suppressive functions in CRC. By regulating the degradation of *TUBB4B* mRNA, PNLDC1 influences cell cycle progression and p53 signaling. The PNLDC1–*TUBB4B* axis represents a novel target for CRC therapy, with MBZ showing promise in reversing the effects of PNLDC1 loss both in vitro and in patient‐derived models. These findings not only enhance our understanding of CRC's molecular underpinnings but also position PNLDC1 as a biomarker and a potential therapeutic target.

## 4 Materials and Methods

### 4.1 Clinical Samples

Matched tumor and adjacent non‐tumor tissues were collected from 91 CRC patients. Prior to sample collection, informed consent was obtained from patients or their relatives. The specimens were promptly frozen in liquid nitrogen and stored at −80°C until use. None of the patients received local or systemic treatments prior to surgery.

### 4.2 Cell Culture

The SW480, HCT116, and HEK293T cell lines were obtained from the American Type Culture Collection (ATCC, USA). HCT116 and SW480 cells were cultured in McCoy's 5A medium (Meilunbio, MA0314) and RPMI‐1640 medium (Sigma, R8758), respectively, while HEK293T cells were cultured in DMEM medium (Sigma, D5546). All media were supplemented with 10% fetal bovine serum (FBS; Yeasen, 40131ES), 100 U mL^−1^ penicillin, and 100 µg mL^−1^ streptomycin (Beyotime, C0222). Cells were maintained at 37°C in a humidified incubator with 5% CO_2_.

### 4.3 *PNLDC1* KO

*PNLDC1* KO in HCT116 cells was achieved using the CRISPR/Cas9 system. Cells were transfected with guide RNA (g1:5′‐TGCTTCACTAGGATGTATGGAGG‐3′, g2:5′‐AAGCTATGGCGTGTTCACTGTGG‐3′), and after 24 h cells were selected with 0.5 µg mL^−1^ puromycin (Beyotime, ST551) for 48 h. Individual clones were isolated and validated by sequencing, RT‐qPCR, and Western blotting.

### 4.4 Lentiviral Infection

HCT116 and SW480 cells in the logarithmic growth phase were seeded into 6‐well plates. After 12 h, the culture medium was replaced with virus suspension (MOI = 15) containing polybrene. After 16 h, the medium was replaced with fresh McCoy's 5A or RPMI‐1640 medium containing 10% FBS. Puromycin (4 µM) was added 48 h post‐infection, and the medium was refreshed every 2 days to remove non‐viable cells. The overexpression efficiencies measured by RT‐qPCR were 600% in HCT116 cells and 1816.7% in SW480 cells.

### 4.5 Colony Formation Assay

Cells (3 × 10^3^ cells/well) were plated in 6‐well plates and cultured for 14 days in a complete medium. Colonies were fixed with 4% paraformaldehyde (Servicebio, G1101) for 10 min, stained with crystal violet (Beyotime, C0121) for 8 min, washed with PBS, and counted by using ImageJ software.

### 4.6 Cell Proliferation Assay

Cell proliferation was assessed using the CCK‐8 kit (Solarbio, CA1210). Cells (8 × 10^3^ cells/well) were seeded in 96‐well plates and incubated at 37°C with 5% CO_2_. At 24, 48, 72, and 96 h, the medium was replaced with 100 µL of 10% CCK‐8 reagent in a serum‐free medium. Absorbance was measured after 1 h using a microplate reader (Molecular Devices, USA).

### 4.7 Transwell Assay

CRC cells (2 × 10^5^ cells/well) in serum‐free medium were seeded into the upper chambers of 8‐µm pore‐size Transwell inserts (Corning, 3422), with 30% FBS as a chemoattractant in the lower chamber. After 48 h at 37°C, cells were fixed with paraformaldehyde (Servicebio, G1101) for 30 min and stained with 0.1% crystal violet (Beyotime, C0121) for 20 min. Non‐migrated cells were removed, and migrated cells were counted under a microscope.

### 4.8 Wound Healing Assay

Cells (1.5 × 10^5^ cells/well) were plated in 12‐well plates. Upon reaching confluency, a wound was made using a 10 µL pipette tip. Medium with 3% FBS was added, and images were captured at 0, 24, 48, 72, and 96 h.

### 4.9 Cell Cycle

A cell cycle staining kit (Multi Sciences, CCS012) was used following the manufacturer's instructions. Cells at 50% confluence were incubated for 24 h, washed with PBS, and stained with Reagent A/B for 30 min at 37°C. Cell cycle distribution was analyzed using a CytoFLEX LX Analyzer (Beckman Coulter, China).

### 4.10 Apoptosis

Cells (1 × 10^6^ cells) were collected and washed with cold PBS. Apoptotic cells were detected using the Annexin V‐Alexa Fluor 488/PI Apoptosis Detection Kit (Yeasen, 40302ES) and analyzed with a CytoFLEX LX Analyzer (Beckman Coulter, China).

### 4.11 RT‐qPCR

Total RNA was extracted using the Hieff qPCR SYBR Green Master Mix (No Rox) kit (Yeasen, 19221ES). cDNA was synthesized with 200 ng of total RNA in a 10 µL reaction volume using transcriptase (Yeasen, 11120ES). RNAs were incubated at 25°C for 5 min, 42°C for 30 min, and 85°C for 5 min and stored at −20°C until RT‐qPCR was performed. The reverse transcription reaction was then diluted to a 30 µL total volume with double‐distilled H_2_O. RT‐qPCR was performed in Hieff UNICON Advanced qPCR SYBR Master Mix (Yeasen, 11185ES) with RT‐qPCR primer sequences (Table S2). Reactions were conducted in triplicate using the CFX96 Touch Real‐Time Detection System (Bio‐Rad, USA), and the program consisted of 2 min at 95°C and then 40 cycles of 95°C for 10 s, 60°C for 30 s followed by a melt curve at 60–95°C for 10 min. The ΔCt values from independent experiments were used to calculate the expression fold change using the 2^−ΔΔCt^ method.

### 4.12 WB Analysis

The total cell lysate was generated by lysing cells in a radioimmunoprecipitation assay (RIPA) buffer (Beyotime, P0013). Lysates were cleared by centrifugation at 12,000 rpm for 5 min (4°C). The clear lysate was mixed with SDS sample buffer (Yeasen, 20315ES). Protein extracts (10 µg per sample) were separated on 12% SDS‐PAGE gels and transferred to PVDF membranes. Membranes were blocked with 5% milk for 1 h and incubated overnight at 4°C with primary antibodies. Blots were visualized using Western‐HRP substrate (Millipore, USA) and detected with the Bio‐Rad ChemiDoc Touch system. Antibody details are listed in Table S1. Bands were normalized to β‐actin.

### 4.13 Cell‐Derived Xenografts

HCT116 cells (1 × 10^7^ cells in 0.2 mL Matrigel (Gibco, A14132)) were injected subcutaneously into the right flank of 4‐week‐old male NCG mice (GemPharmatech, China). For MBZ (MedChemExpress, HY17595) treatment, when tumors reached 100 ± 10 mm^3^, mice were randomized into vehicle and MBZ (50 mg kg^−1^) groups. MBZ were administered via oral gavage once daily, 5 days per week. Tumor length (*L*) and width (*W*) were measured with Vernier calipers, and tumor volume was calculated with the formula (*L* × *W* ^2^)/2. Mice were sacrificed via cervical dislocation when the tumor diameter reached 20 mm in any group of mice for analysis.

### 4.14 RIP

RIP assays were performed following established protocols [75]. Ten million cells were collected and lysed in RIP buffer (20 mM Tris‐HCl pH = 7.4, 100 mM KCl, 1.5 mM MgCl_2_, 0.2 mM EDTA, 1% NP‐40, 100U RNase inhibitor, 1 × cocktail), and lysates were incubated with 6 µg anti‐PNLDC1 antibody‐coated beads (Proteintech, 25559‐1‐AP) overnight at 4°C. Immunocomplexes were washed with washing buffer for 3 times (20 mM Tris‐HCl pH = 7.4, 100 mM KCl, 1.5 mM MgCl_2_, and 0.2 mM EDTA), treated with proteinase K, and RNA was extracted using phenol/chloroform/isoamyl alcohol (125:24:1). Clean reads were aligned to the hg38 genome using bowtie2 (v2.5.1). Both input and RIP samples were prepared for next‐generation sequencing. RNA library standard: Total RNA content >1 µg concentration >20 µg µL^−1^; OD260/280: 1.7–2.5; OD260/230: 0.5–2.5, RIN >6; 28s/18s:1–5. RNA Clean reads were aligned to the hg38 genome using bowtie2 616 (v2.5.1). Both input and RIP samples were prepared for next‐generation sequencing by genomic consistency analysis was conducted by Beijing Biomarker Technologies Co. Ltd. (Beijing, China). RIP‐seq raw data are uploaded to GEO database, GEO Submission (GSE287213).

### 4.15 RNA Sequencing Data Processing and Analysis

Raw sequencing data were processed using fastp v0.23.4 to filter out low‐quality and low‐complexity reads and remove adapter sequences [76]. Cleaned reads were aligned to the hg38 reference genome in paired‐end mode using bowtie2 (v2.5.1) [77]. The resulting BAM files were converted to bigWig format using bamCoverage from deeptools (v3.5.3) [78] for chromosome coverage visualization. Custom R scripts were utilized to generate chromosome coverage plots.

Metagene plots were created using computeMatrix and plotProfile functions from deeptools (v3.5.3). Peak calling was performed with MACS2 (v2.2.9.1) [79] using the callpeak function, and highly reproducible peaks across replicates were identified using IDR (v2.0.4.2) [80]. After quality control checks, data from replicates were merged to generate a combined set of peaks for each group.

The overlap and intersection of peaks between cell lines were assessed using Venn diagrams generated by the intersect function in bedtools (v2.27.1) [81]. Motif analysis was conducted with MEME (v5.0.2) [82], restricting motif length to 6–10 bp and retaining motifs with significant E‐values (<10^−3^). Peak annotation, including overlapping gene locations, was performed with bedtools (v2.27.1) and reference gene annotation data from encode regions. Functional annotation of genes was done using the DAVID online database (https://david.ncifcrf.gov/; last accessed October 2023), focusing on cell cycle, apoptosis, and transcription regulation‐related genes. Genomic distribution of peaks was visualized using IGV (v2.16.2) [83].

### 4.16 Immunofluorescence

Cells or tissues were fixed in 4% paraformaldehyde (Servicebio, G1101) for 30 min at room temperature (for cell cultures) or overnight at 4°C (for tissues), washed with PBS for 3 times and blocked with blocking buffer (Beyotime, P0102). The samples were incubated with primary antibodies for 2 h at room temperature (cells) or 24 h at 4°C (tissues). Then, the samples were incubated with secondary antibodies (1:500) for 30 min at room temperature. Samples were imaged with an Olympus confocal microscopy. Antibody details are listed in Table S1.

### 4.17 HCR

On the first day, samples were fixed with 4% paraformaldehyde (Servicebio, G1101) and washed with PBST 5 times for 5 min. Hybridization buffer (50% formamide, 5 × sodium chloride sodium citrate, 9 mM citric acid pH = 6.0, 0.1% Tween‐20, 50 µg mL^−1^ heparin, 1 × Denhardt's solution, 10% dextran sulphate) and primers were added, and samples were incubated at 37°C overnight. Second day: washing with 300 µL probe wash buffer (50% formamide, 5 × sodium chloride sodium citrate, 9 mM citric acid pH = 6.0, 0.1% Tween‐20, 50 µg mL^−1^ heparin) 3 times for 15 min at 37°C, followed by washing with 1 mL 5 × sodium chloride sodium citrate and 0.1% Tween‐20 at room temperature 3 times for 15 min. Then amplification was performed by incubating samples with amplification buffer (5 × sodium chloride sodium citrate, 0.1% Tween‐20, 10% dextran sulphate) and amplifier (Sangon Biotech, China) at room temperature overnight. Third day: washing with 1 mL 5 × SSCT (0.75 M NaCl, 0.075 M Sodium citrate, 0.1% Tween‐20) 5 min for 2 times, then washing with 1 mL 5 × SSCT 30 min for 2 times, then washing with 1 mL 5 × SSCT for 5 min. Then samples were imaged with an Olympus confocal microscope.

### 4.18 Cell Division Assay

Prior to fixation, cells were synchronized using 2 mM thymidine block for 17 h followed by release into fresh medium for 8 h. To enrich for mitotic cells, cultures were subsequently treated with 10 ng mL^−1^ nocodazole for 5 h to depolymerize microtubules. Following this treatment, cells were fixed and subjected to dual immunofluorescence staining.

### 4.19 Co‐IP

Fresh CRC tissues from surgical resection was snap‐frozen in liquid nitrogen and stored at −80°C until use. Fifty milligram CRC tissue were disrupted in 1 mL lysis buffer (20 mM Tris‐HCl pH = 7.4, 100 mM KCl, 1.5 mM MgCl_2_, 0.2 mM EDTA, 1% NP‐40, 100U RNase inhibitor, 1 × cocktail). After centrifugation, supernatants were incubated with 6 µg anti‐TUBB4B antibody (Santa Cruz, sc‐134230) or 6 µg anti‐p53 antibody (Proteintech, 20683‐2‐Ig)‐coated beads respectively overnight at 4°C. Immunocomplexes were washed with washing buffer for 3 times (20 mM Tris‐HCl pH = 7.4, 100 mM KCl, 1.5 mM MgCl_2_, 0.2 mM EDTA) and analyzed by WB.

### 4.20 PTCs Testing

Biopsy samples were collected and processed by Zhejiang Jiji Precision Medicine Co., Ltd. (China). PTCs were generated and treated with MBZ. Drug response data were provided within 2 weeks of biopsy collection, following protocols from prior research [32].

### 4.21 Statistical Analysis

Data were analyzed using GraphPad Prism 9 and presented as mean ± s.d. Statistical significance was determined using Student's *t*‐test, with *p*‐values < 0.05 considered significant. All Western blot and dot–blot images are representative of three independent experiments.

### AI Statement

The authors used DeepSeek‐V4 for language editing purposes only. No AI tools were employed in data analysis, figure preparation, or the generation of scientific content. All authors have reviewed and take full responsibility for the final manuscript.

## Author Contributions

Methodology: L.L., Y.G., Z.M., W.Y., Z.X., Z.C., and F.Y. Software: L.S., T.Z., and Z.L. Resources: Y.Q and G.C. Funding acquisition: X.Y, X.C, and L.H. Writing: L.L,X.L, and S.O. Supervision: X.C, Y. Y, and L.H.

## Ethical Approval Statement

All animal experiments were approved by the Animal Experimentation Ethics Committee of Zhejiang University (Approval No. ZJU20230105). All participating patients were informed and written informed consent was obtained from all participants. All procedures involving human subjects were conducted in accordance with the principles of the Declaration of Helsinki. All patient‐related experiments were approved by the Institutional Ethical Board of the Zhejiang Chinese Medical University (Project No. 2024‐KL‐023‐01).

## Funding

This study was supported by the National Natural Science Foundation of China (No. 32271311 and No. 82200784).

## Conflicts of Interest

The authors declare no conflicts of interest.

## Supporting information




**Supporting File 1**: exp270222‐sup‐0001‐SuppMat.docx.

**Supporting File 2**: exp270222‐sup‐0002‐TableS4.xlsx.

## Data Availability

The FASTQ files and processed data for bulk RNA‐seq reported in this paper were deposited to the Gene Expression Omnibus database under accession numbers GSE287213.
