# Supplementary material for: ER‐Localized Deadenylase PNLDC1 Suppresses Colorectal Cancer Progression by Targeting the mRNA Decay of TUBB4B
Source: Exploration (Beijing). 2026 Sep 21;6(5):70222. doi: 10.1002/exp2.70222 (PMC13593926; doi:10.1002/exp2.70222)
Supplement: Supplementary file 1 [file EXP2-6-70222-s001.docx]

Extended Information

**ER-Localized Deadenylase *PNLDC1* Suppresses Colorectal Cancer Progression by Targeting the mRNA decay of *TUBB4B***

Inventory of extended information

Extended Data Fig.s and Tables

Fig. S1 is related to Fig. 1.

Fig. S2 is related to Fig. 2.

Fig. S3 is related to Fig. 3.

Fig. S4 is related to Fig. 4.

Fig. S5 is related to Fig. 5.

Fig. S6 is related to Fig. 6.

Fig. S7 is related to Fig. 7.

Table S1 is related to Materials and Methods.

Table S2 is related to Materials and Methods.

Table S3 is related to Materials and Methods.

**Extended Data Fig. 1**

(A) *PNLDC1* expression in CRC tumor tissues (*n* = 275) compared with normal tissues (*n* = 349) based on GEPIA online analysis. (B) Correlation between *PNLDC1* methylation and TMB score in CRC (*n* = 393). (C) Correlation between CRC metastasis and *PNLDC1* mRNA expression in CRC, M0 (*n* = 387) and M1 (*n* = 67). (D) Overall survival of CRC patients with low PNLDC1 and high PNLDC1 expression (*n* = 35). (E) Expression of PNLDC1 across CRC pathological stages: I (*n* = 9), II (*n* = 29), III (*n* = 21), and IV (*n* = 17). (F) PNLDC1 expression analysis based on tumor position: Colon (*n* = 41) and Rectum (*n* = 25). (G) Correlation analysis on the expression of PNLDC1 and tumor volume (*n* = 69). (H) PNLDC1 expression analysis based on pathological classification: Mucinous carcinoma (*n* = 5) and Adenoma (*n* =58). (I) PNLDC1 expression analysis based on gross classification: infiltrative (*n* = 6), ulcerative (*n* = 34), and protruded (*n* = 28).

**Extended Data Fig. 2**

(A) mRNA relative expression of *PNLDC1* in several colorectal cancer cell lines. (B) *PNLDC1* knockout (KO) using CRIPSR/Cas9. (C) Western blot of *PNLDC1* KO by using HCT116 cell line. (D) Western blot of *PNLDC1* KD by using HCT116 cell line. (E) Quantification of colony formation assay in Fig. 2B. (F) *PNLDC1* KO-C10 in HCT116 cells increased cell proliferation demonstrated by CCK-8 assays. (G) Immunofluorescence staining for M-phase HCT116 cells (phospho-H3^+^) and mitotic spindles (α-tubulin) and quantification of M-phase HCT116 cells (phospho-H3^+^) with multipolar spindles (Scale: 5 μm). (H) Quantification of Transwell assay in Fig. 2C. (I) RT-qPCR results of *PNLDC1* expression in vector and overexpression (OE)-*PNLDC1* HCT1116 cells. (J) Quantification of Transwell assay in Fig. 2F. (K) RT-qPCR results of *PNLDC1* expression in vector and OE-*PNLDC1* SW480 cells. (L) Western blot of PNLDC1 OE by using SW480 cell line. (M) Transwell migration assays were performed to examine the migration of OE-*PNLDC1* and normal SW480 cells. (N) Scratch assays revealed the OE of *PNLDC1* retarded the migration of SW480 cells (100 μm scale). (*n* = 3)

**Extended Data Fig. 3**

(A) mRNA relative expression of *PNLDC1* in several kinds of human cell lines (A549, BT474, HepG2, HepG3, HCT116, MRC5, SKOV3, HeLa, HEK293T, PC-3, AGS). (B) Quantification of PNLDC1 colocalization with ER-marker protein by Pearson’s correlation coefficient (*n* = 3). (C) Typical fluorescence confocal image of overexpression GFP-PNLDC1 in low expressed HEK293T cell lines.

**Extended Data Fig. 4**

(A) Deadenylase activity of the microsome fractions isolated from the HEK293T cells by the size-exclusion chromatography (SEC) assay. The number of total proteins in each fraction was kept the same as determined by the bovine serum albumin (BSA) kit. (B) PNLDC1-binding motifs and representative RNAs in HEK293T cells. (C) PNLDC1-binding motifs and representative RNAs in HCT116 cells. (D) RT-qPCR results of PNLDC1-binding RNA expression in paracancer and cancer tissue in patients. (E) Alterations of ER stress pathway-related proteins expression under *PNLDC1*-KO. (F) ATF4 and CHOP expression in xenograft tumors injected with control versus *PNLDC1*-KO cells (*n* = 3). (G) GSEA enrichment plot for "Regulation of ER Unfolded Protein Response" pathway. (H) Enriched sequence motifs identified PNLDC1-binding sites of *ATF4* mRNA. (I) Decay kinetics of *ATF4* mRNA measured by quantitative RT-qPCR, showing reduced degradation rates under *PNLDC1*-KO.

**Extended Data Fig. 5**

(A) Correlation between *PNLDC1* mRNA expression and *TUBB4B* mRNA expression from http://timer.comp-genomics.org. (B) Correlation between *PNLDC1* mRNA expression and *TUBB4B* mRNA expression from linkdomics portal (*n* = 379). (C) Correlation between *PNLDC1* mRNA expression and *TUBB4B* mRNA expression from cBio portal (*n* = 592). (D) HCR analysis shows increased *TUBB4B* mRNA levels in OE*-PNLDC1* cells (*n* = 21) compared to controls (*n* = 38) (scale: 5 μm). (E) IF of TUBB4B of paracancer and tumor tissues from CRC patients. (*n* = 6, scale: 20 μm).

**Extended Data Fig. 6**

(A) PPI network analysis of TUBB4B using the STRING database, indicating potential interactions with key cell cycle proteins. (B**)** Western blot revealed MBZ had no effect on the expression of TUBB4B. (C) Images of tumor isolated from HCT116 *PNLDC1*-KO subcutaneous implantation treated with vehicle and MBZ. (D) Quantification of cell cycle in Fig. 6E (*n* = 3). (E) Quantification of WB in Fig. 6J (*n* = 3). (F) Expression of tubulin isoforms following *PNLDC1* KD and MBZ treatment. (G-H) Quantification of WB in Extended Data Fig. 6F (*n* = 3). (I) Dose-response curves of HCT116 cells treated with cisplatin for 48 hours (*n* = 3). (J) The combinatorial effect of MBZ and cisplatin on cell proliferation assessed by CCK-8 assay (*n* = 3).

**Extended Data Fig. 7**

(A) Intraoperative photograph of CRC patients. (B) Viability of PTCs treated with low concentration of mebendazole (*n* = 3). (C) Quantification of cell area in Fig. 7F and extended data Fig. 7D (100 μm scale) (*n* = 3). (D) Images of PTCs treated with MBZ 0 day, 2 days and 7days at concentrations of 1.563 μm, 6.25μm, and 25 μm (100 μm scale).

| Antibodies | Source | Identifier | Dilution |
| --- | --- | --- | --- |
| PNLDC1 | Proteintech | 25559-1-AP | 1:1000 WB; 1:200 IF |
| β-actin | HUABIO | R1207-1 | 1:1000 WB |
| TUBB4B | HUABIO | ET1609-48 | 1:1000 WB; 1:200 IF |
| TUBB4B | Santa cruz | Sc-134230 | IP |
| P53 | Cell Signaling | 2527 | 1:1000 WB; 1:200 IF |
| P53 | Proteintech | 60283-2-Ig | IP |
| P21 | Cell Signaling | 2947 | 1:1000 WB |
| CDK2 | HUABIO | ET-1602-6 | 1:1000 WB |
| CYCLIN E2 | HUABIO | M0407-15 | 1:1000 WB |
| Ki67 | Solarbio | K007523P | 1:200 IF |
| ɑ-tubulin | Proteintech | 66031-1-Ig | 1:20000 WB;1:500 IF |
| β-tubulin | HUABIO | ET1602-4 | 1:20000 WB |
| γ-tubulin | HUABIO | ET1702-32 | 1:1000 WB |
| TUBB3 | HUABIO | M0805-8 | 1:2000 WB |
| pH3 | Cell Signaling | 3377 | 1:800 IF |

**Table S1. Antibody information**

**Table S2. Sequence of primers for RT-qPCR**

| Gene name | Primers | Sequence |
| --- | --- | --- |
| *PNLDC1* | Forward | TGGGAAGGGAGTTGCATACG |
|  | Reverse | CAAAGGCAGCCAACCTGGTA |
| *β-actin* | Forward | GGCTGTATTCCCCTCCATCG |
|  | Reverse | CCAGTTGGTAACAATGCCATGT |
| *TUBB4B* | Forward | GGACAACTTCGTTTTCGGTCA |
|  | Reverse | CCTTTCTCACAACATCCAGCAC |
| *PARP16* | Forward | CCTCAAAGGTCCTGACAATCC |
|  | Reverse | CTAGGCGGCTACCATGAAATG |
| *NPM1* | Forward | ACGGTCAGTTTAGGGGCTG |
|  | Reverse | CTGTGGAACCTTGCTACCACC |
| *MAP3K20* | Forward | TTTTGGGAGTGTTTATCGAGCC |
|  | Reverse | CAATGCCATAGTTGGGAGGTTC |
| *FBXL12* | Forward | CACTATGCCCAAGCTCAGAGT |
|  | Reverse | GACGATGACCATACAGTGGGG |
| *AMBRA1* | Forward | GCCCAGACAGGACTCTCTTAG |
|  | Reverse | TGAACACACTTGCCAGTCTTC |
| *TOPBP1* | Forward | TTCAGCAACTCACAGTTAAGCA |
|  | Reverse | GGCACACTCATACTTCTGACC |
| *IER1* | Forward | CAGCCGCAGGGTTCTCTAC |
|  | Reverse | GATCTGGCAGAAGACGATGGT |

**Table S3. Sequence of primers for si*TUBB4B***

| Gene name | Primers | Sequence |
| --- | --- | --- |
| *TUBB4B* | Forward | CCAGACAGGAUCAUGAACA (dT) |
|  | Reverse | UGUUCAUGAUCCUGUCUGG (dT) |
